# Attention-deficit/hyperactivity disorder with developmental coordination disorder: 24-year follow-up of a population-based sample

**DOI:** 10.1186/s12888-021-03154-w

**Published:** 2021-03-22

**Authors:** Valdemar Landgren, Elisabeth Fernell, Christopher Gillberg, Magnus Landgren, Mats Johnson

**Affiliations:** 1 Gillberg Neuropsychiatry Centre, Sahlgrenska Academy, Gothenburg University, Kungsgatan 12, Gothenburg, 411 19 Sweden; 2 Department of Psychiatry, Skaraborg Hospital, Lövängsvägen, Skövde, 54142 Sweden

**Keywords:** ADHD, Affective disorders, Neuropsychiatry

## Abstract

**Background:**

Although the body of research concerning neurodevelopmental disorders is vast, there is a scarcity of longitudinal studies beyond late adolescence, and of studies taking co-existing disorders into account. The present study aimed to investigate outcome in adulthood for children with attention-deficit/hyperactivity disorder (ADHD) combined with developmental coordination disorder (DCD) diagnosed at 6.6 years of age.

**Methods:**

Out of a screening-based population cohort of 589 individuals, 62 (10 female) diagnosed with ADHD+DCD at mean age 6.6 years naïve to stimulant treatment were followed into adulthood through national registries. Results were compared to a screen- and assessment negative population matched group from the same cohort (PM group, *n* = 51) and a registry-matched (RM group, *n* = 410) group of the same county and age.

**Results:**

At 30 to 31 years of age, five deaths had occurred; one in the ADHD+DCD group and two each in the comparison groups. In time to event analyses of the composite outcome of any psychiatric disorder, psychotropic prescription, sick pension or criminal sentence, events occurred at a significantly higher rate in the ADHD+DCD group (*p* = 0.0032, vs PM group *p* = 0.0115, vs RM group *p* = 0.0054). The ADHD+DCD group had significantly higher rates of psychiatric diagnoses, prescriptions of psychoactive medications and occurrence of sick pension than both comparison groups. Further, the ADHD+DCD group had significantly lower educational attainment compared to both comparison groups, more years with unemployment, and overall higher welfare recipiency. Rates of pain diagnoses and analgesic prescriptions did not separate the groups.

**Conclusion:**

ADHD+DCD entailed a less favorable outcome in adulthood compared to a non-clinical comparison group and a registry-matched population. Neurodevelopmental disorder diagnosed upon school entry is of prognostic utility with respect to function in adulthood, and warrants early identification and management.

**Supplementary Information:**

The online version contains supplementary material available at 10.1186/s12888-021-03154-w.

## Background

Although it is now well established that individuals with neurodevelopmental disorders often meet diagnostic criteria for more than one disorder, this fact has generally not been reflected in longitudinal outcome studies of attention-deficit/hyperactivity disorder (ADHD) in adulthood [1]. Outcome studies have commonly focused on ADHD severity and measures of intelligence, but overlooked other important co-occurring symptoms and neurodevelopmental diagnoses that may contribute to prognosis. Thorough neurodevelopmental assessments will almost always detect impairing symptoms from multiple disorders, more so with repeated assessments across the life span [2]. Such clinical observations have been corroborated by family- twin- and genetic studies wherein a wide array of neurodevelopmental symptoms co-aggregate [3–5].

With a view to emphasizing this coexistence of disorders, the concept of ESSENCE was launched by Gillberg [2]. ESSENCE, the acronym for Early Symptomatic Syndromes Eliciting Neurodevelopmental Clinical Examinations, embraces all kinds and severities of neurodevelopmental disorders (NDD), e.g., intellectual disability, autism spectrum disorder, ADHD, developmental coordination disorder (DCD) language disorders, specific learning disorders, Tourette syndrome and early onset epilepsies. The ESSENCE concept highlights the importance of considering all functional areas and describing the individual’s total amount of functional deficits and disabilities. The concept also emphasizes the need for etiological concerns. This holistic view would create the best possible conditions for follow-up and intervention.

Most long-term outcome studies of ADHD report an association with lower academic and occupational achievement, psychiatric comorbidity, substance use/abuse, risk of accidents and relationship problems [1, 6]. Putative predictors for long-term outcome include IQ, conduct problems, symptom severity and parental factors such as psychopathology and parental practices [6]. These factors (or predictors) were also highlighted in a meta-analysis of ADHD-persistence into adulthood by Faraone et al. [7], wherein eight studies followed participants into their twenties, and only one study into their thirties. Besides the relatively short follow-up time beyond early adulthood, included studies have been further limited by clinic-based sampling bias and by failure to account for effects of comorbid problems (e.g. autism spectrum disorder was considered an exclusion criterion for ADHD according to DSM-III and IV).

DCD is characterized by a delay in the development of gross and fine motor skills, poor motor planning and coordination, resulting in difficulties or inability to acquire common, everyday skills dependent on integration of executive, cognitive and emotional processes [8].

Although recognition of DCD varies with diagnostic practices across countries, when ascertained it occurs in a severe form in 5% of children [9, 10], and can be considered a marker of attention deficits [9, 11, 12], autistic traits [9, 12] and poorer reading skills [10, 13], as well as of depressive symptoms [11] persisting into teenage years.

Among very few studies reporting on the trajectory of DCD into adulthood, a recent prospective cohort study of women with autism and/or ADHD revealed that one in four had a history of DCD in addition to ADHD and/or ASD. In this group chronic pain was reported by 77% [14]. Aligned with studies engaging patients with a chief complaint of pain, both retrospective studies of adults [15], and cross-sectional studies of children [16] report increased rates of neurodevelopmental disorder symptoms of which ADHD has been most commonly assessed.

### Aims of the study

In addition to describing the outcome in adulthood of ADHD with coexisting DCD, the aim was also to prospectively test whether an association to pain of unknown etiology and prescription of analgesic medications can be replicated.

## Methods

### Study overview

The original study was initiated in 1992 with a view to validating a physician-led school-entry screening for NDD and to establish the rate of such disorders in a population-based sample of children [17, 18], Results yielded a minimum prevalence rate of 10.7% for NDD and a prevalence of 5.3% for the combination of ADHD+DCD specifically [17]. A simplified motor examination had a sensitivity of 80% and specificity of 100% of detecting ADHD+DCD at school entry and predicted poorer academic achievement at 9 years of age [13, 17, 18].

We now conducted a registry-based follow-up study 24 years later of the group with ADHD+DCD and the original comparison group with no NDD, with data sources tracking participants from birth until 2017 (i.e., at 30 or 31 years of age).

### Definitions

Based on research of minimal brain dysfunction (MBD) in the 1970’s [19, 20], the diagnosis of “deficits in attention, motor control and perception” (DAMP) was defined as the combination of: (1) cross situational impairing attention deficit (ADD), with or without impairing hyperactivity/impulsivity; and (2) impairing deficit in at least one of the following areas: gross motor, fine motor, perception, i.e. the experience and interpretation of sensory information, or speech-language, in the absence of intellectual disability and/or cerebral palsy/other major neurological impairment. Severe DAMP was diagnosed in cases showing the combination of (1) and all of the deficits listed under (2) [21]. It was widely used throughout Scandinavia at the time of inception of the original study and in subsequent publications [13, 17, 18].

With the increasing clinical use of DSM-III-R and DSM-IV, DAMP as a diagnostic entity was supplanted by attention-deficit/hyperactivity disorder (ADHD), conferring clinical focus on the reported behavioral component and a gradual decline of neuromotor examination of children. The operationalized definition of DAMP is equivalent to ADHD with coexisting developmental coordination disorder (DCD) as defined in DSM-IV and its subsequent revisions [21]. This has been corroborated by follow-up studies of DAMP, where all participants met criteria for ADD according to DSM-III, and at least 85% met the DSM-IV criteria for ADHD, in addition to DCD [22]. However, it should be noted that the clinical gestalt of DAMP in its severe form (including all deficits listed under (2) above) entails more impairment than explained by ADHD and DCD “only”.

### Participants

**Fig. 1:**
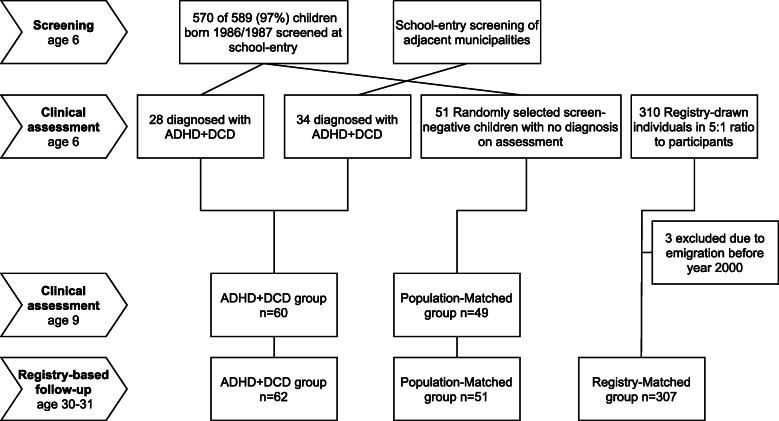
Participant Flow Diagram

A participant flow diagram is depicted in Fig. 1. The original study was conducted in a rural municipality of Western Sweden with a median income similar to the country average. Between 1992 and 1994, parents of 570 of 589 (97%) children born 1986–1987 had agreed to participate in a population-based epidemiological study. The 570 children were screened for NDD upon school entry (mean age 6.6 y). Neurodevelopmental disorders comprised DAMP, motor perception dysfunction (equivalent to DCD), ADHD, Tourette syndrome, mental retardation, cerebral palsy, autism and autistic-like disorder). Diagnoses were ascertained in clinical assessments by a multidisciplinary team in 63 (10.7%) participants, of which ADHD with coexisting DCD was found in 28 [17]. Through screening of adjacent municipalities 34 participants with ADHD+DCD were consecutively recruited, yielding a total of 62 participants (52 male, 10 female) diagnosed with coexisting ADHD and DCD (Fig. 1). A population matched comparison group (PM group) of 51 participants (39 male, 12 female) randomly selected from screen-negative children of the same municipality and who were assessed negative for NDD in an identical procedure was recruited [18]. The socioeconomic distribution of the PM group was identical to a cross-sectional sample of the general Swedish population at the time, and anthropometric measurements (height, weight and head circumference) were similar in the ADHD+DCD and PM group [23].

We conducted a registry-based follow-up in adulthood, as this minimizes risk of attrition and improves comparability of results. To increase statistical power and avoid type-I errors we added a registry-matched comparison group (RM group, *n* = 310) in a 5:1 ratio to the index cohort (ADHD+DCD, *n* = 62, PM group, *n* = 51). To minimize bias (e.g. immortal time and ascertainment bias) the RM group was drawn at age 7 from the general population in the same county (adjacent municipalities covered by the same hospital system) and matched for sex (Fig. 1). We argued that significant findings consistent in comparisons of the ADHD+DCD group with both RM and PM groups, would less likely be spurious.

### Outcomes

Outcomes were constructed with data from four national registers; the National Patient Register (NPR), the Prescribed Drug Register (PDR), the Longitudinal Integration Database for Health Insurance and Social Studies (LISA; 2001–2017) and the National Crime Register. Utilizing unique personal identification numbers assigned to all citizens at birth, Statistics Sweden (the national agency holding the LISA-register) linked all sources and provided de-identified individual-level data. Data was collected for the period 1993 to 2017 (Study inclusion at age 7 to age 30 or 31). Clinically meaningful endpoints were constructed in accordance with review articles and other registry-based follow-up studies [7, 24–26]. A prior study of ADHD+DCD in a Swedish context with face-to-face follow-up in young adulthood found poorer outcome (defined as one or more of sick pension, substance use disorder, criminal sentence, any psychiatric disorder with significant impairment) among those with ADHD+DCD (58% vs 13% in the comparison group) [22]. Although our registry-based follow-up did not allow for direct replication, we constructed a similar composite outcome, defined as one or more of (i) any psychiatric disorder, (ii) psychotropic medication prescription, (iii) sick pension or (iv) criminal sentence.

#### Social and demographic outcomes

From the LISA-register we obtained data on grades, education, occupation, welfare benefits due to sick pension, sick leave, social circumstances or unemployment, and family demographics. The register integrates yearly sociodemographic data of all citizens 15 years or older since 1990. Education level is reported on a 7-point ordinal scale; 1 = less than 9 years, 2 = 9 years, 3 = 1–2 years of high school, 4 = 3 years of high school, 5 = 1–2 years of undergraduate college, 6 = 3 or more years of undergraduate college, 7 = graduate studies. Occupational skill level is reported according to International Standard Classification of Occupation on a 5-point ordinal scale; 0 = No vocation reported in the register (never worked, or never on a permanent contract), 1 = Manual work not requiring education beyond elementary school level (Newspaper distributors, fast food staff, freight handling), 2 = Requiring education programmes at upper secondary and tertiary level of no more than 2 years (Carpenter, industry production, retail service, assistant nurse) 3 = Requiring practical or vocational tertiary education programmes of 2–3 years in length (Banking officials, technician, chef, police), 4 = Requiring theoretical and research-oriented tertiary education programmes and third-cycle programmes of at least 3 years, normally 4 years or longer in length (Physician, nurse, teacher, civil engineer).

#### Criminality

The national crime register covers all lower court sentences in Sweden since 1973. Crime was defined here as the occurrence of a sentence. Violent crime was defined in accordance with previous registry-based studies as “homicide and attempted homicide, aggravated assault (an assault that is life-threatening in nature or causes severe bodily harm), common assault, robbery, threatening behavior, harassment, arson, and any sexual crime” [26]. Drug-related crime was defined as a drug-related primary sentence, including driving under influence. Number of crimes included in each sentence is reported in the register, but only the primary crime was used.

#### Prescriptions

The prescribed drug register provided data on all medications dispensed from a pharmacy since 2005 (99% coverage). Psychotropic drugs were defined as class N01-N07 according to the Anatomic Therapeutic Chemical classification system (ATC).

#### Diagnoses

From the national patient register, we retrieved all ICD-10 (1997–2017) diagnoses from inpatient (99% coverage) and outpatient care (since 2001, 70–96% coverage) [27, 28]. The register covers pediatric, internal medicine, surgery and psychiatric health care services, but primary care providers are not included. Psychiatric diagnoses were defined as F1-F9 according to ICD-10. To test the possible association of ADHD+DCD to pain, we collapsed the ICD-10 diagnoses with a chief complaint of pain of unknown etiology (G43 Migraine, G44 Tension headache, K30 Functional dyspepsia, M25 Joint pain, M54 Back pain, M79 Myalgia, M94 Other disorders of cartilage, O26 Pregnancy pain, R07 Chest pain, R10 Stomach pain, R51 Headache, R52 Unspecific pain.

#### Ethics

Caregivers signed informed consent on behalf of their children in the original study. Swedish law states that research of interest for patients with similar ailments as that being studied may not require explicit consent, if it poses minimal risks to the individual and is unattainable by other reasonable means. A waiver of consent was therefore approved from the regional ethics review board of Gothenburg university (Dnr 172–18) for the study’s registry-based follow-up with deidentified data.

### Statistics

Sample sizes were defined a priori and we considered it sufficient to replicate findings from a previous follow-up study (*n* = 55 + 46), reporting significantly poorer outcome among those with ADHD+DCD (58% vs 13% in the comparison group) [22]. We used two-tailed tests and α = 0.05. Binary data of proportions were analyzed as groupwise pairwise comparisons with χ^2^-tests. To utilize data efficiently by taking both occurrence and timing of events into account, binary data was analyzed as time-to-event and depicted in Kaplan-Meier curves whenever appropriate. The starting point was defined as year of birth, and the event by the binary variable at study. Participants were right-censored at death or emigration, and thus all participants with any data on the outcome could be included. If the log-rank test for differences between all three groups was significant, we did pairwise log-rank tests of cases vs RM and PM groups with *p*-value adjustment according to Bejamini and Hochberg [29]. Kaplan-Meier curves were depicted in accordance with Morris et al. [30], along with cumulative rates of events and censoring in a risk table.

Due to modest group sample sizes, we assumed non-normal distributions and used non-parametric analyses for both ordinal and continuous variables. If Kruskal-Wallis rank tests indicated statistical significance, pairwise comparisons were done with Mann-Whitney U tests for the ADHD + DCD group vs each comparison group separately. To explore the weight of outcome events, we analyzed quantitative aspects (e.g. sum of welfare recipiency received, days in unemployment, prescribed daily dose of medication) specifically among those experiencing the particular outcome. Based on Lipsey et al. [31] we converted results to Cohens’ *d* (i.e. standardized mean difference) to make results more interpretable in some instances. Descriptive data, statistical tests and visualizations were performed with R 3.6.3 (R Core team 2020), the *KMunicate* [32], *ggplot2* [33] and *esc* [34] packages.

## Results

**Fig. 2:**
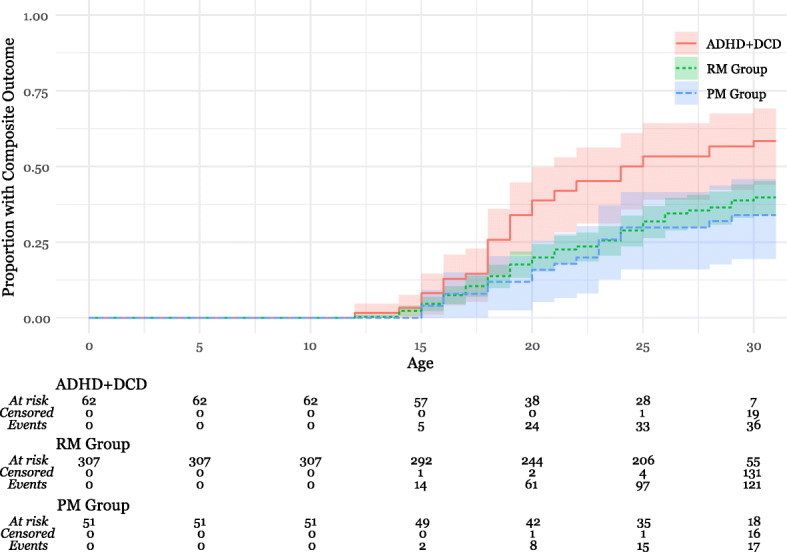
Kaplan-Meier Curve of Composite Outcome (Criminal Conviction, Sick Pension, Psychiatric Disorder or Psychotropic Medication Prescription). Legend: Kaplan-Meier curve depicting time to the event and proportion of participants in each group experiencing the event. “At risk” is the number of participants partaking in the study who have not experienced the event, “Censored” is the number of participants exiting the study due to emigration or death, and “Events” is the number of participants experiencing the outcome. Abbreviations: Attention-Deficit hyperactivity Disorder with Developmental Coordination Disorder (ADHD+DCD), Registry Matched group (RM Group), Population Matched Group (PM Group)

**Fig. 3:**
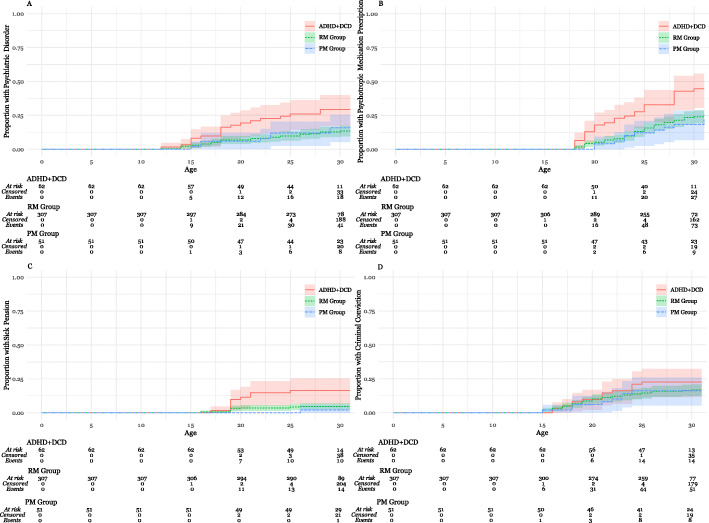
**a-d**. Kaplan-Meier Curves of Outcomes in Adulthood. Legend: Kaplan-Meier curve depicting time to the event and proportion of participants in each group experiencing the event. “At risk” is the number of participants partaking in the study who have not experienced the event, “Censored” is the number of participants exiting the study due to emigration or death, and “Events” is the number of participants experiencing the outcome. Abbreviations: Attention-Deficit hyperactivity Disorder with Developmental Coordination Disorder (ADHD+DCD), Registry Matched group (RM Group), Population Matched Group (PM Group)

The 420 participants (ADHD+DCD *n* = 62, PM group *n* = 51, RM group *n* = 307, 342 male 77 female) were followed to a mean age of 30 years (range 15–31), in total contributing a sum of 9693 person-years (mean 23, min 8, max 24) of follow-up time since study inclusion at age 7. In the 10 instances of missing data beyond 25 years of age, reasons were death (*n* = 5) and emigration (*n* = 5). As demonstrated in Fig. 2, Kaplan-Meier analyses of the composite outcome of any psychiatric disorder, psychotropic medication prescription, sick pension or criminal sentence, showed that events occurred at a significantly higher rate in the ADHD+DCD group (Fig. 2, logrank test *p* = 0.0032, vs PM *p* = 0.0115, vs RM *p* = 0.0054). In separate analyses (Kaplan-Meier curves in Fig. 3 A-D), differences were significant for receiving a psychiatric disorder (logrank test *p* = 0.005, vs PM group *p* = 0.12, vs RM group *p* = 0.004), psychotropic prescription (*p* = 0.0009, vs PM group *p* = 0.0086, RM group *p* = 0.0014) and sick pension (*p* = 0.0007, vs PM *p* = 0.0176, vs RM *p* = 0.0017) and not significant for criminal convictions (*p* = 0.53).

### Demographic data and criminality

**Fig. 4:**
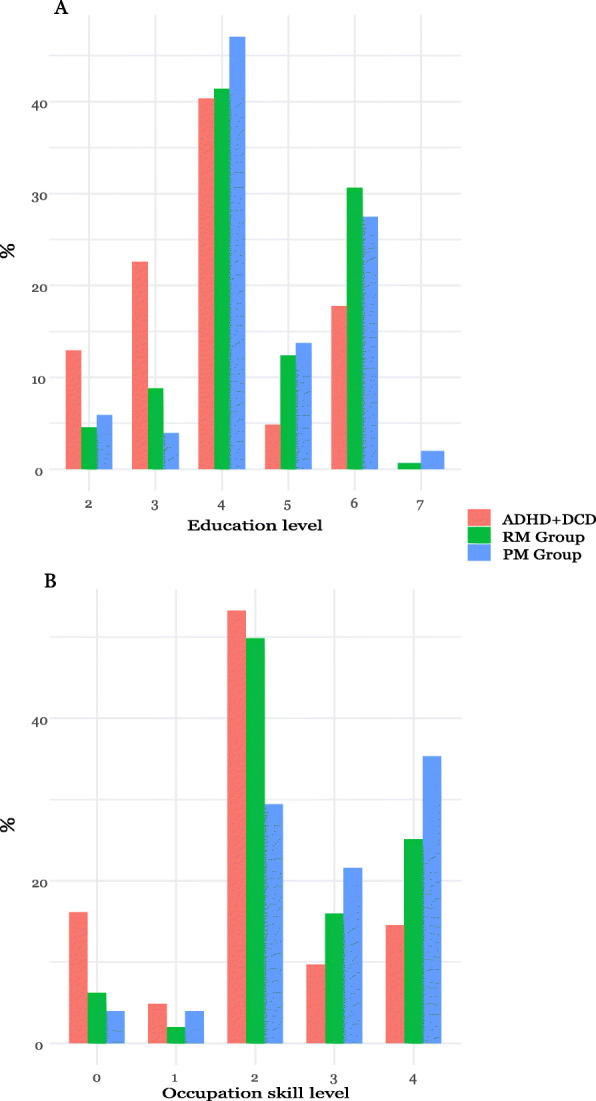
**a-b**. Education and Occupation Skill Level Distributions. Legend: **a**: Education level: 1 = less than 9 years, 2 = 9 years, 3 = 1–2 years of high school, 4 = 3 years of high school, 5 = 1–2 years of undergraduate college, 6 = 3 or more years of undergraduate college, 7 = graduate studies. Missing data; ADHD+DCD (1), RM group (3). **b**: International Standard Classification of Occupation (See methods section for a detailed description); 0 = No vocation, 1 = Manual work not requiring education beyond elementary school level, 2 = Requiring education programmes at upper secondary and tertiary level of no more than 2 years, 3 = Requiring education programmes of 2–3 years in length, 4 = Requiring theoretical and research-oriented education. Missing data; ADHD+DCD (1), PM (3), RM (3). Abbreviations: Attention-Deficit hyperactivity Disorder with Developmental Coordination Disorder (ADHD+DCD, *n* = 62), Registry Matched group (RM Group, *n* = 307), Population Matched Group (PM Group, *n* = 51)

Social and demographic data is reported in Table 1. As illustrated in Fig. 4, the ADHD+DCD group had significantly lower educational attainment and occupational skill level distribution compared to both comparison groups (Table 1, educational attainment, ADHD+DCD vs RM Cohens *d* = − 0.71, 95% Confidence Interval [CI − 0.33 to − 1.09], vs PM -0.55 [95% CI − 0.18 to − 0.93], occupational skill level, vs RM Cohens *d* = − 0.60 [95%CI − 0.22 to − 0.98], vs PM *d* = − 0.71 [95%CI − 0.33 to − 1.10]). Further, they were numerically less likely to be parents or married, but not significantly so. Compared to the PM group, participants with ADHD+DCD had significantly more days in unemployment (*d* = 0.41 [95% CI 0.03 to 0.78]) and more years with occurrence of unemployment (*d* = 0.55 [95% CI 0.17 to 0.93]). The occurrence welfare recipiency (due to unemployment, sick pension, sick leave or social circumstances) in the group with ADHD+DCD was significantly higher than for both comparison groups (Table 1, logrank test *p* = 0.0002, vs PM *p* = 0.00040, vs RM *p* = 0.00067, Kaplan-Meier curves are provided in the additional file), and the total sum of welfare benefits among recipients was significantly higher among those with ADHD+DCD compared to the PM group (Table 1, *d* = 0.64 [95% CI 0.26 to 1.02]).

**Table 1:** Outcomes in Adulthood

Demographic and social outcomes	ADHD+DCD (*n* = 62)	Registry-Matched Group (*n* = 307)	Population-Matched Group (*n* = 51)	ADHD+DCD vs Registry Matched Comparison Cohens *d*	ADHD+DCD vs Population Matched Comparison Cohens *d*
Female, no (%)	10 (16)	55 (18)	12 (24)	–	–
Ever married, no (%)^1^	12 (19)	68 (22)	15 (29)	N.S	N.S
Being a parent, no (%)^1^	25 (37)	145 (47)	26 (49)	N.S	N.S
Education level, range 1–7, mdn (IQR)^2^	4 (3–4)	4 (4–6)	4 (4–6)	**−0.71 (− 0.33 to − 1.09)**	**− 0.55 (− 0.18 to − 0.93)**
Sports grade 9th grade failed or missing, no (%)	10 (16)	32 (10)	2 (4)	0.28 (− 0.15 to 1.73)	**0.87 (0.00 to 1.73)**
Occupational skill level, range 0–4, mdn (IQR)^3^	2 (2–2)	2 (2–4)	3 (2–4)	**− 0.60 (− 0.22 to − 0.98)**	**− 0.71 (− 0.33 to − 1.10)**
Occurrence of unemployment, no (%)^4^	45 (73)	187 (61)	29 (57)	**–**	**–**
*Among participants with unemployment*					
No of years receiving any unemployment benefit, mdn (IQR)	3 (2–6)	3 (2–6)	2 (1–3)	0.11 (− 0.26 to 0.49)	**0.41 (0.03 to 0.78)**
Average no. of days per year in unemployment as adult, mdn (IQR)	15 (9–27)	14 (7–30)	8 (5–17)	0.09 (−0.28 to 0.47)	**0.55 (0.17 to 0.93)**
Occurrence of sick leave, no (%)^4^	26 (42)	109 (36)	21 (41)	–	**–**
*Among participants with sick leave*					
Income per year (SEK) from sick leave, mdn (IQR)	2200 (700 to 5800)	2300 (700 to 5400)	1200 (700 to 2500)	N.S	N.S
No of years with any sick leave, no (IQR)	1 (1–2)	1 (2–2)	1 (1–1)	0.31 (−0.06 to 0.68)	0.18 (− 0.19 to 0.55)
Occurrence of welfare benefits due to social circumstances^4^	16 (26)	57 (19)	7 (14)	–	–
Occurrence of any welfare benefits^4^	52 (84)	198 (65)	29 (57)	–	–
*Among participants receiving welfare benefits*					
Sum of any welfare benefits^1^ (SEK) received per year from 18 years, mdn (IQR)	8600 (3700 to 21,300)	9300 (2400 to 17,800)	900 (1600 to 8700)	0.20 (−0.17 to 0.57)	**0.64 (0.26 to 1.02)**
Occurrence of welfare benefits in the last year of follow-up^1^ (age 30 or 31), no (%)	16 (28)	64 (21)	9 (19)	N.S	N.S
Any conviction^4^ no, (%)	14 (23)	51 (17)	8 (16)	–	–
Violence	10 (16)	30 (10)	4 (8)	–	–
Drug-related	3 (5)	9 (3)	1 (2)	–	–
Traffic-related	3 (5)	23 (7)	4 (8)	–	–
Other crime	1 (2)	11 (4)	1 (2)	–	–
**Psychiatric and medical oucomes, no (%)**					
Deceased^6,7^	1 (2)	2 (1)	2 (4)	N.S	N.S
Hospital admission for any cause^4^	35 (57)	176 (57)	21 (41)	–	–
Psychiatric hospital admission^7^	2 (3)	1 (0)	0 (0)	N.A	N.A
Support by Social Services Act as child (SOL)^7^	5 (8)	6 (2)	0 (0)	N.A	N.A
Support by Support and Service for Persons with Certain Functional Impairments (LSS)^7^	4 (6)	7 (2)	0 (0)	N.A	N.A
Any psychiatric disorder^8^	18 (29)	41 (13)	8 (16)		
Affective disorders	6 (10)	14 (5)	3 (6)	–	–
Anxiety disorders	4 (6)	19 (6)	4 (8)	–	–
Neurodevelopmental disorders	12 (19)	11 (4)	1 (2)	–	–
Substance use related disorders	2 (3)	12 (4)	3 (6)	–	–
Pain diagnosis^4,9^	21 (34)	76 (25)	12 (24)		
*Prescriptions*					
Psychotropic medication prescription^4^	27 (44)	74 (24)	10 (20)		
Stimulants	6 (10)	6 (2)	0 (0)	–	–
Antidepressant	17 (27)	50 (16)	8 (16)	–	–
Anxiolytics	14 (23)	45 (15)	4 (8)	–	–
Antiepileptics	6 (10)	15 (5)	1 (2)	–	–
Sedatives	7 (11)	25 (11)	5 (10)	–	–
Neuroleptics	3 (5)	10 (3)	1 (2)	–	–
Anti-alcohol medication	0 (0)	0 (0)	0 (0)	–	–
Anti-opioid medication	0 (0)	0 (0)	0 (0)	–	–
*Among participants with prescription*					
Total amount of defined daily dose psychotropic medication prescribed, mdn (IQR)	433 (79 to 1971)	356 (101 to 1925)	171 (63 to 2172)	N.S	N.S
Any pain medication prescription^4^, no (%)	30 (48)	128 (41)	20 (39)		
Opioids, no (%)	14 (23)	67 (22)	10 (20)	N.S	N.S
*Among participants with pain medication prescription*					
Total amount of defined daily dose of analgesic medication prescribed, mdn (IQR)	54 (38 to 123)	50 (24 to 100)	50 (23 to 67)	N.S	N.S

*Abbreviations*: *Mdn* median, *IQR* Interquartile range, *SEK* Swedish Krona. Point estimates of Cohens *d* (i.e. standardized mean difference) is reported with the 95% confidence interval in parentheses. Bolded numbers are significant

^1^ The 3 × 2 χ^2^ test was not significant and therefore no pairwise analyses or effect size calculations were performed

^2^ Education level: 1 = less than 9 years, 2 = 9 years, 3 = 1–2 years of high school, 4 = 3 years of high school, 5 = 1–2 years of undergraduate college, 6 = 3 or more

years of undergraduate college, 7 = graduate studies. Missing data: ADHD+DCD (1), Registry-Matched group (RM group, 3)

^3^ International Standard Classification of Occupation; 0 = No vocation, 1 = Manual work not requiring education beyond elementary school level, 2 = Requiring education programmes at upper secondary and tertiary level of no more than 2 years, 3 = Requiring education programmes of 2–3 years in length, 4 = Requiring theoretical and research-oriented education. Missing data; ADHD+DCD (1), Population-Matched group (3), RM group (3)

^4^ Outcome was analyzed as time-to-event in a Kaplan-Meier curve and is provided in the additional file

^5^ Welfare benefits due to sick leave, sick pension, unemployment or social circumstances

^6^ Causes of death included accident, suicide and cardiac arrest, but cannot be attributed in detail due to participant identity protection

^7^ Distributions violated the assumptions for the χ^2^ test and no effect sizes were calculated

^8^ Corresponding ICD-10 codes for diagnoses; Any psychiatric disorder (F1-F9), Affective disorders (F3), Anxiety disorders (F4), Neurodevelopmental disorders (F7, F82, F84, F90), Substance use disorders (F1)

^9^ ICD-codes diagnoses with a chief complaint of pain of unknown etiology are provided in the methods section

Causes of sick pension in the ADHD+DCD group (*n* = 10) were most likely Autism Spectrum Disorder (ASD) + ADHD (*n* = 3), ASD only (*n* = 3), ADHD (*n* = 2), Epilepsy (*n* = 1) and unknown (*n* = 1). No significant differences were found across the three groups in occurrence of welfare recipiency due to unemployment (*p* = 0.2), sick leave (*p* = 0.68), social circumstances (*p* = 0.27), any criminal conviction (*p* = 0.53, Fig. 3), violent crime (*p* = 0.28), traffic-related crime (*p* = 0.54), or drug-related crime (*p* = 0.64), (Kaplan-Meier curves are provided in the additional file).

### Prescriptions and health care utilization

As reported in Table 1, five deaths occurred (One in the ADHD+DCD group, two each in the comparison groups). Three participants were admitted to a psychiatric hospital, two in the ADHD+DCD group (unspecified affective disorder and mania) and one in the RM group (depressive episode). The proportion with hospitalization for any cause were similar across groups (χ^2^-test N. S, Table 1). There were no instances of venereal disease in the ADHD+DCD or PM group and five instances in the RM group. Among psychiatric disorders in pediatric or psychiatric specialty care, neurodevelopmental disorders were most frequent in the ADHD+DCD group (Table 1). Of those in the ADHD+DCD group with registered NDD:s (*n* = 12), five were diagnosed with ADHD, three with ASD and four with ADHD and ASD. In the RM group (*n* = 11), five were diagnosed with ASD, three with ADHD and three with intellectual disability. In the PM group one participant was diagnosed with ADHD. There were no registered diagnoses of DCD (ICD-code F82). No suicide attempts or prescriptions of medications for substance use disorders (anti-opioid or anti-alcohol medication) were registered in any group. Among the psychotropic prescriptions, stimulants were prescribed at significantly higher rates (*p* = 0.0012, vs PM *p* = 0.036, vs RM *p* = 0.004, see Kaplan Meier curve in Additional file 1). Differences were not significant regarding pain diagnoses (*p* = 0.25), accidents (*p* = 0.86), prescriptions of anxiolytics (*p* = 0.06), antidepressants (*p* = 0.09), antiepileptics (*p* = 0.14), neuroleptics (*p* = 0.63), sedatives (*p* = 0.69), analgesics (*p* = 0.58) or opioids (*p* = 0.93), although a nominally higher rate of events generally occurred in the ADHD-DCD group (Kaplan-Meier curves are provided in the additional file).

## Discussion

In this prospective cohort study of long-term outcomes, the diagnosis of ADHD+DCD made after a general population screening at school entry was associated with increased rates of sick pension, welfare dependence, psychiatric disorder, and prescription of psychotropic medications in adulthood. At the time of the initial study a diagnosis in the study did not confer referral to care, because specific interventions for ADHD+DCD (e.g. parental programs, stimulant treatment) were almost non-existent in the area. Hence, because only 25% of those with ADHD+DCD received a formal clinically registered diagnosis of NDD (e.g. ADHD or ASD) in primary pediatric or psychiatric care services, and a minority (10%) was prescribed stimulants after 2005, we consider the study to mainly portray the natural course of the “syndrome”.

In comparisons, results generally exhibited a uniform direction of increase or decrease, with the PM group at the better end, RM in between, and ADHD+DCD group at the poorer end. When the uniform direction was present but differences were statistically insignificant, this may likely have been due to insufficient power rather than being a true null-result.

In terms of education and occupational proficiency, the ADHD+DCD group exhibited a shift toward lower attainment. This is consistent with a recent Danish report of completed educational grades in final high-school examinations, wherein ADHD was associated with both fewer completed grades and lower grades (Cohens *d* − 0.62) [25]. Out of 629,622 children in the Danish cohort, 38,001 (6%) had a mental disorder, and 4% had ADHD. In contrast, DCD, with an expected prevalence of 5%, was diagnosed in 0.02% of children. Although participants had co-existing disorders diagnosed (number of diagnoses is almost twice as many as the number of participants), the role of co-occurring impairments was not accounted for, nor discussed. While the large registry data set provides power and statistical precision, the study by Dalsgaard et al. also highlights two important limitations of registry-based studies. First, relying on regular care diagnoses of neurodevelopmental disorders confer systematic bias due to poor recognition of many conditions, as illustrated by the low rate of DCD in their cohort. Second, this limitation makes inferences of the relative contribution from neurodevelopmental disorders on outcome unreliable. Thus, there is still a need for in-depth clinical studies to better gauge the role of co-existing factors.

The proportion reported to have no occupational skill (ADHD+DCD 16%, PM 6%, RM 4%, Fig. 2) could be due to only working on short-term contracts (for which subjects are not reported as permanently hired and visible in the registry), but is in light of low rates in the comparison groups most parsimoniously explained by *absence* of any significant work experience. Further, participants differ strikingly in terms of average recipiency of welfare benefits in adulthood, where median sums are almost 10 times higher in the ADHD+DCD group compared to the PM group. In contrast, the occurrence of welfare recipiency in the last year of follow-up showed no meaningful difference (Table 1). Therefore, rather than the occurrence per se, it is the weight of impairment and need for welfare support *over time* that singles out the ADHD+DCD group. This can be conceived as adult endpoints of the childhood impairments apparent in academic, sports and everyday tasks associated with DCD [10, 13].

Groups did not differ meaningfully in rates of accidents or hospitalizations, (Table 1, Kaplan-Meier curve in Additional file), contrary to previous studies of ADHD showing elevated risks for accidents [35]. We think that the phenotype with predominately attention deficit and coexisting DCD more likely subsume to a sedentary lifestyle, in which impulsivity is not a hallmark. This is in line with childhood studies of DCD, wherein participants are at risk of obesity and non-participation in vigorous physical activity [36].

Rates of medication prescriptions in the comparison groups are on par with a recently reported national Swedish male conscript cohort of similar age span (*N* = 414,595) [24]. On the contrary, the ADHD+DCD group had significantly more prescriptions of psychotropic medications, and nominally higher rates of prescriptions for virtually all subclasses (Table 1, Kaplan-Meier curves in Additional file). Besides stimulants, the increase was most pronounced in the class of antidepressants and anxiolytics, drugs with a primary indication of anxiety and depression. Subtracting the somewhat lower rates of *registered diagnoses* (register did not cover primary care) of affective disorders from these prescriptions (Table 1), it can be inferred that participants with ADHD+DCD in adulthood were diagnosed with anxiety and depression in primary care. This is in line with previous reports on an association between DCD and affective symptoms in early adolescence and suggests persistence into adulthood [11]. The rates of pain diagnoses and analgesic prescriptions did not differ significantly between groups, although the ADHD+DCD group had the highest rates of both. A longer follow-up period may give a more comprehensive picture. For instance, fibromyalgia was diagnosed at a mean age of 37 years in the study by van Rensburg et al. [15]. Additionally, there may be sex differences in the magnitude of this association. Asztély et al. reported chronic pain in 77% of adult women with ADHD and/or ASD [14], and both of these studies predominately included women, whereas only 19% were women in our study.

When comparing results to the only previous follow-up study of ADHD+DCD conducted at 22 years of age [22], differences across groups are similar, but less pronounced in this study. For example, rates of criminal offences and indices of substance use did not differ meaningfully across groups in this study, whereas it was evident in their cohort, together with even more pronounced differences in educational attainment [22]. Because drug use in Sweden is less common in rural settings, we hypothesize that county differences (i.e. previous study conducted in an urban area, and the current in rural municipalities) may partly explain the lower rates of substance use in our cohort [37]. Rates of sick pension were similar, as were the proportion with no poor outcome in the composite measure (around 40%). This is consistent with the notion that a substantial minority of those with ADHD as children will have few symptoms and little impairment as adults [1]. Barbaresi et al. reported on adult outcome of a population-based cohort with and without ADHD, recruited by retrospective case ascertainment through medical records [38]. Of 367 participants, 63% (*n* = 232) participated in a clinical follow-up at a mean age of 27 years. Similarly, no disorder was diagnosed at adult follow-up in 38% of participants with ADHD in childhood. ADHD persisted in 38%, of which 57% had a co-occurring psychiatric disorder (in descending frequency, alcohol abuse, antisocial personality disorder, substance abuse, hypomanic episodes, anxiety and depression), compared with 35% of controls. However, differences in measurement methods both at study inclusion (screening and clinical assessment vs retrospective case ascertainment) and follow-up (registry data vs clinical assessment) precludes definite conclusions, especially regarding psychiatric characteristics. Positive predictive value of childhood NDD:s in the NPR is generally good (> 80%) in recent years [39, 40]. But the discrepancy between registered diagnoses of ADHD in the RM group (0.3%) and the expected rates reported in research studies of ADHD in adulthood (~ 3%) indicates that NDD:s for the period at study largely were undetected in clinical practice, and a low negative predictive value for adulthood NDD:s in the NPR for this time period [41]. In light of aforementioned limitations with reliance on registry data only, we think our results provide a minimum level description of adverse outcome for the group with ADHD+DCD.

Strengths of the study includes the population-based sampling, providing the full panorama of the condition at study, the long follow-up time, valid comparison groups and low attrition rate. However, the study is somewhat limited by the modest sample size of the index cohort with ADHD+DCD. This did not allow for meaningful analyses subgrouping ADHD with or without DCD, and made inferences on rare, but important events (such as severe criminality, suicide or death) impossible. Absence of face-to-face assessments precludes information from subtle clinical findings or repeated neuropsychiatric assessments, physical exam and the experiences reported from participants themselves. Apart from prescription medications, data sources are lacking on the study populations utilization of primary care. Ideally, longitudinal studies combine registry data and clinical assessments for comprehensive profiling of participants.

In conclusion, ADHD+DCD diagnosed at school-entry was associated with a less favorable outcome in early adulthood compared both to matched peers without NDD and a registry-drawn group from the same county. The objective nature of neuromotor function tests opens up the possibility that children at risk of adverse outcome in adulthood might be detected prior to school-entry and receive proper support early in education.

## Supplementary Information

**Additional file 1.**

## Data Availability

The datasets used and/or analysed during the current study are available from the corresponding author on reasonable request.
