# Supplementary material for: Attention-deficit/hyperactivity disorder with developmental coordination disorder: 24-year follow-up of a population-based sample
Source: BMC Psychiatry. 2021 Mar 22;21:161. doi: 10.1186/s12888-021-03154-w (PMC7983399; doi:10.1186/s12888-021-03154-w)
Supplement: Supplementary file 1 — Additional file 1. [file 12888_2021_3154_MOESM1_ESM.docx]

# Attention-Deficit/Hyperactivity Disorder with Developmental Coordination Disorder: 24-year Follow-up of a Population-Based Sample

Running head: ADHD with DCD: 24-year follow-up

Valdemar Landgren*^1^, Elisabeth Fernell^1^, Christopher Gillberg^1^, Magnus Landgren^1^, Mats Johnson^1^

1. Gillberg Neuropsychiatry Centre, Sahlgrenska Academy, Gothenburg University, Gothenburg, Sweden.

**Social Outcomes Figure 1**
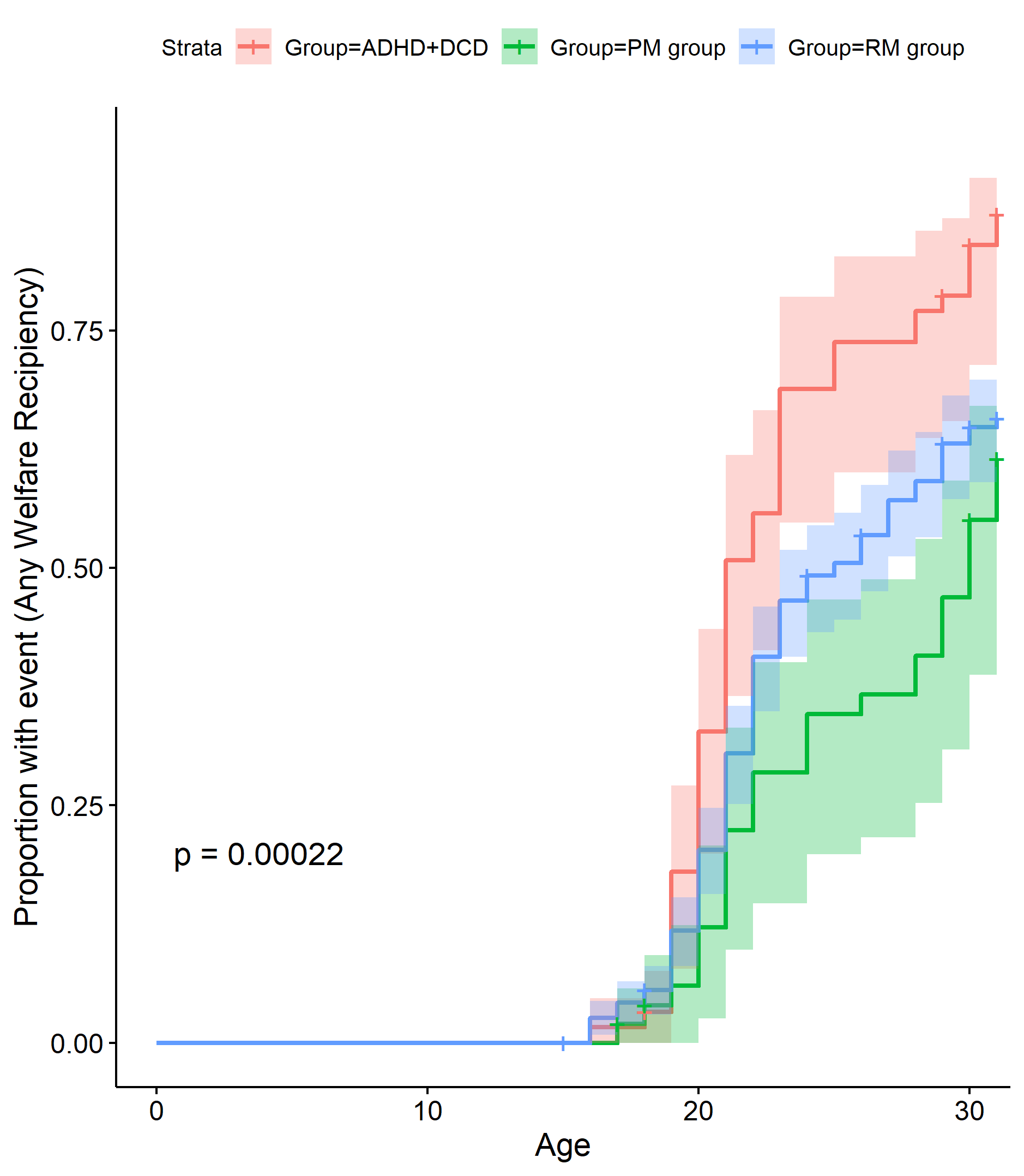


Depicted are Kaplan-Meier curves and overall significance of logrank test of difference across the three groups; ADHD+DCD (n=62), Population Matched group (PM group, n=51) and Registry Matched group (RM group, n=307). Censoring was due to emigration or death.

**Social Outcomes Figure 2**
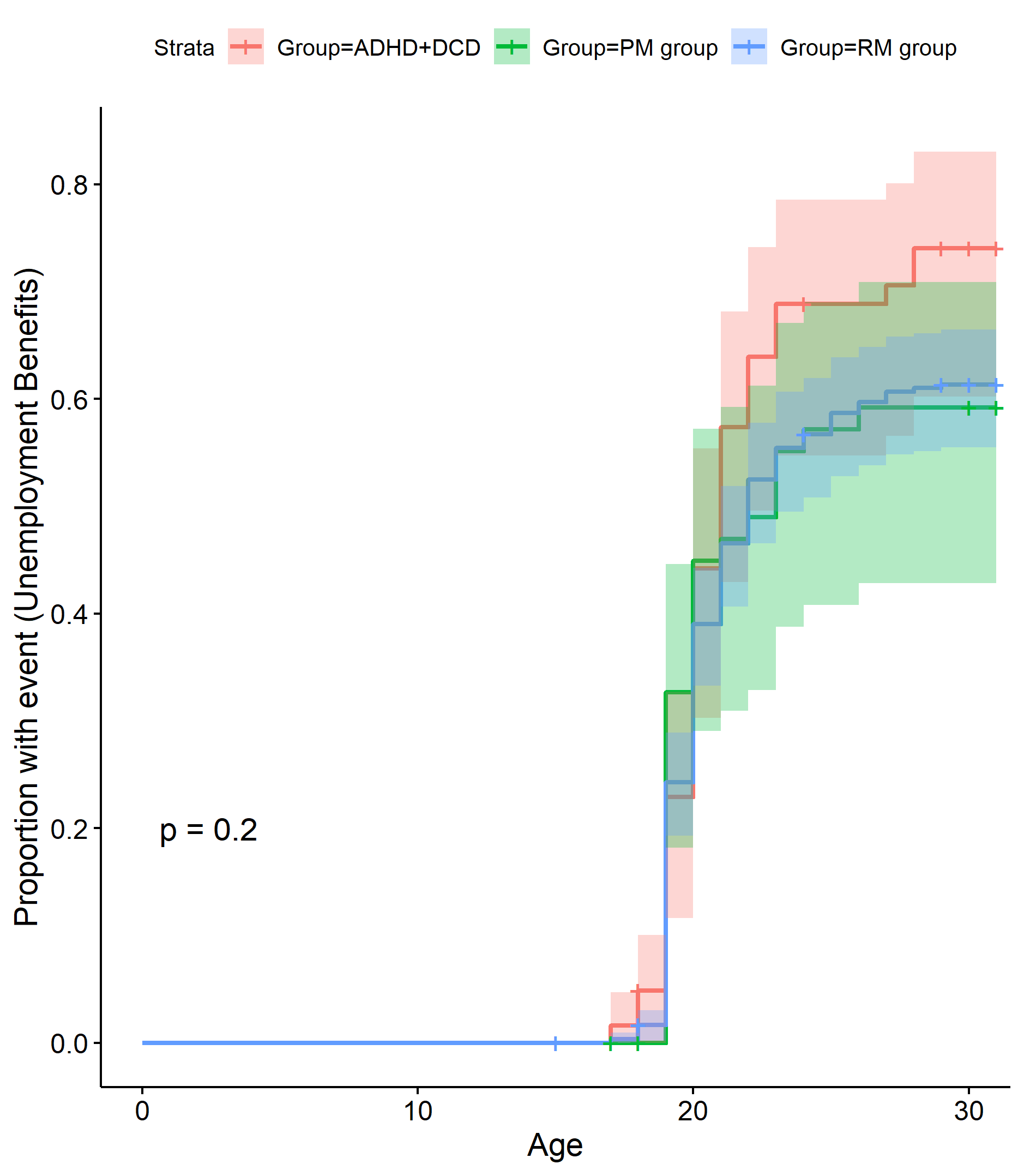


Depicted are Kaplan-Meier curves and overall significance of logrank test of difference across the three groups; ADHD+DCD (n=62), Population Matched group (PM group, n=51) and Registry Matched group (RM group, n=307). Censoring was due to emigration or death.

**Social Outcomes Figure 3**
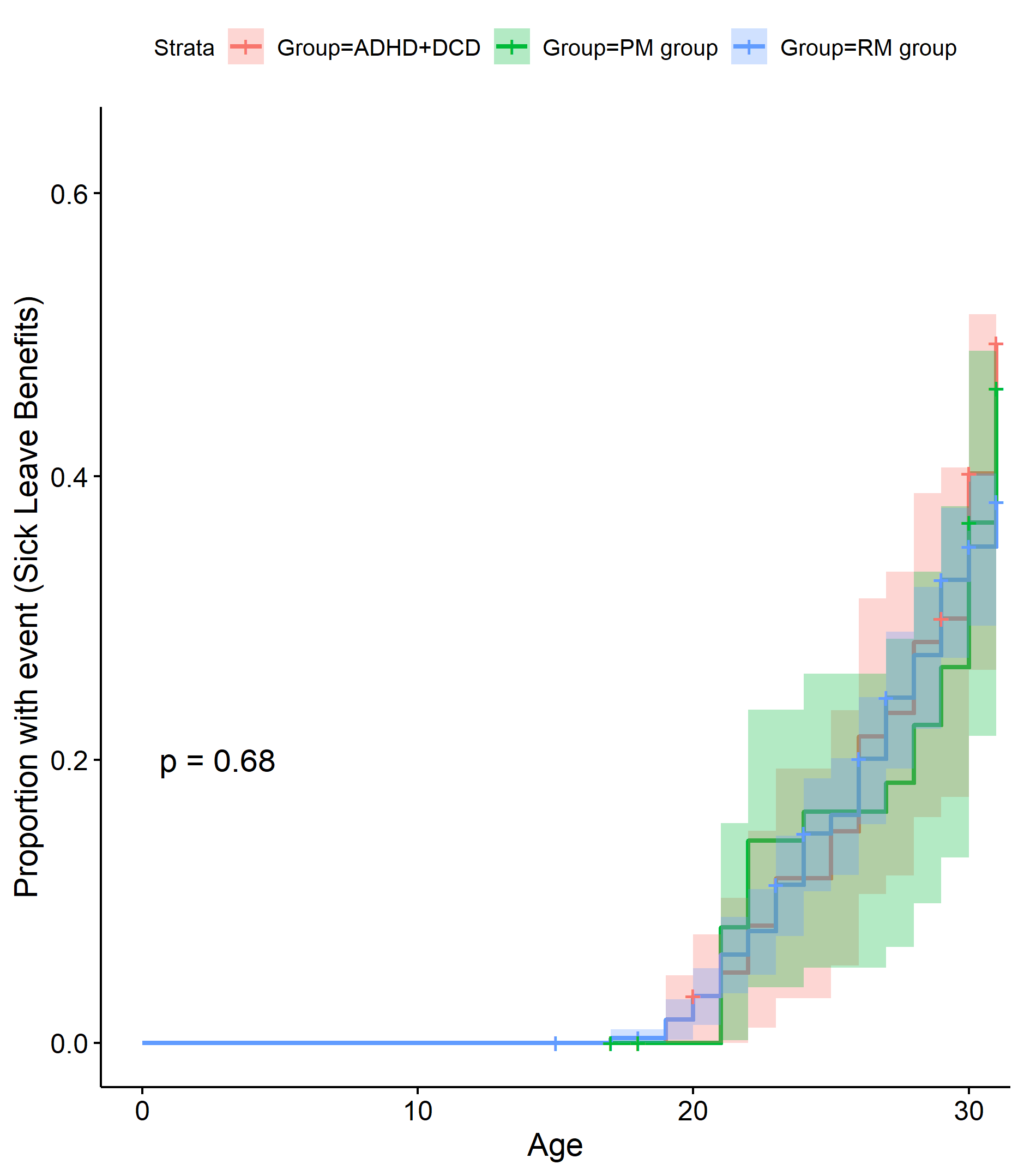


Depicted are Kaplan-Meier curves and overall significance of logrank test of difference across the three groups; ADHD+DCD (n=62), Population Matched group (PM group, n=51) and Registry Matched group (RM group, n=307). Censoring was due to emigration or death.

**Social Outcomes Figure 4**


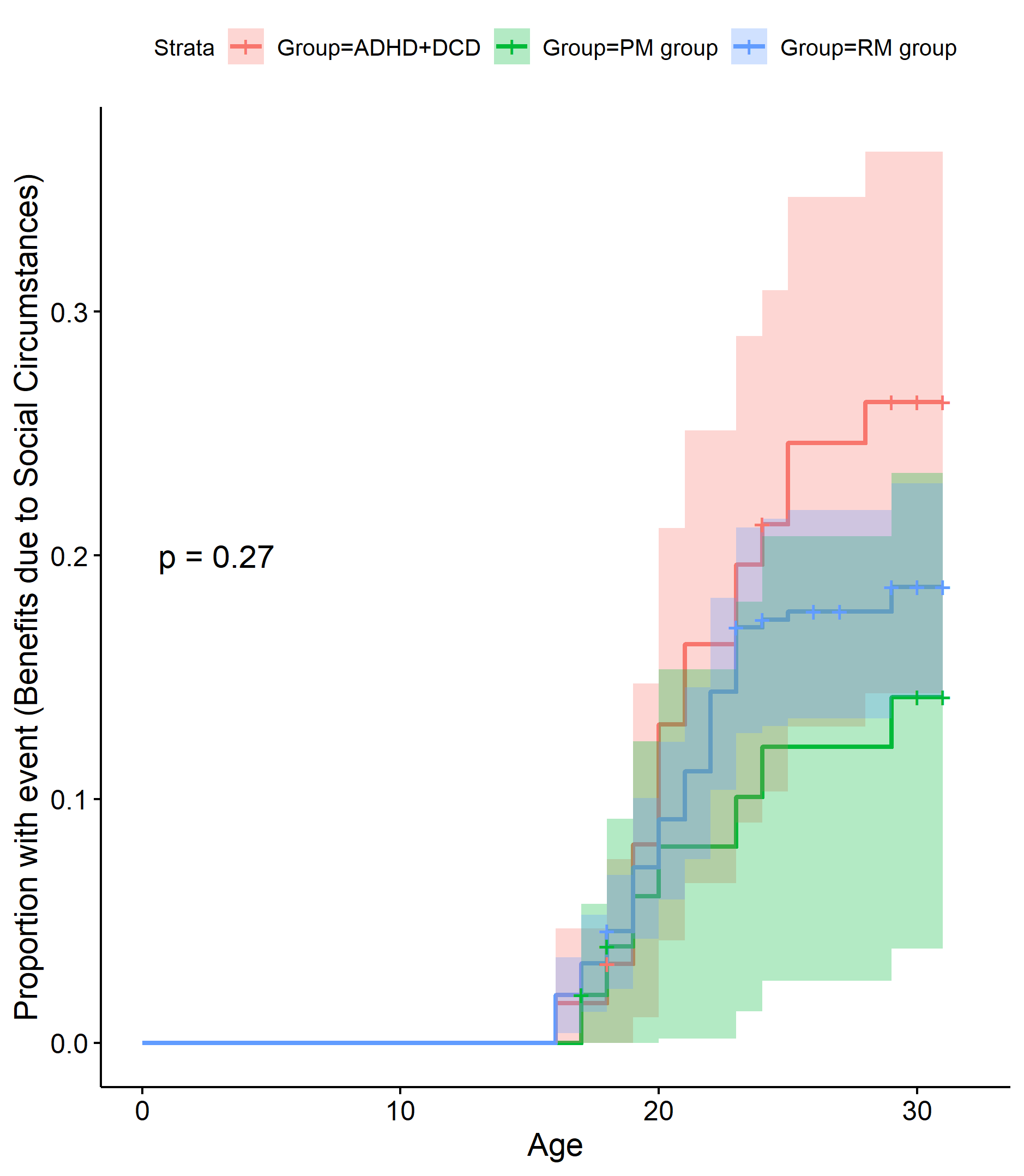


Depicted are Kaplan-Meier curves and overall significance of logrank test of difference across the three groups; ADHD+DCD (n=62), Population Matched group (PM group, n=51) and Registry Matched group (RM group, n=307). Censoring was due to emigration or death.

**Criminality Figure 1**


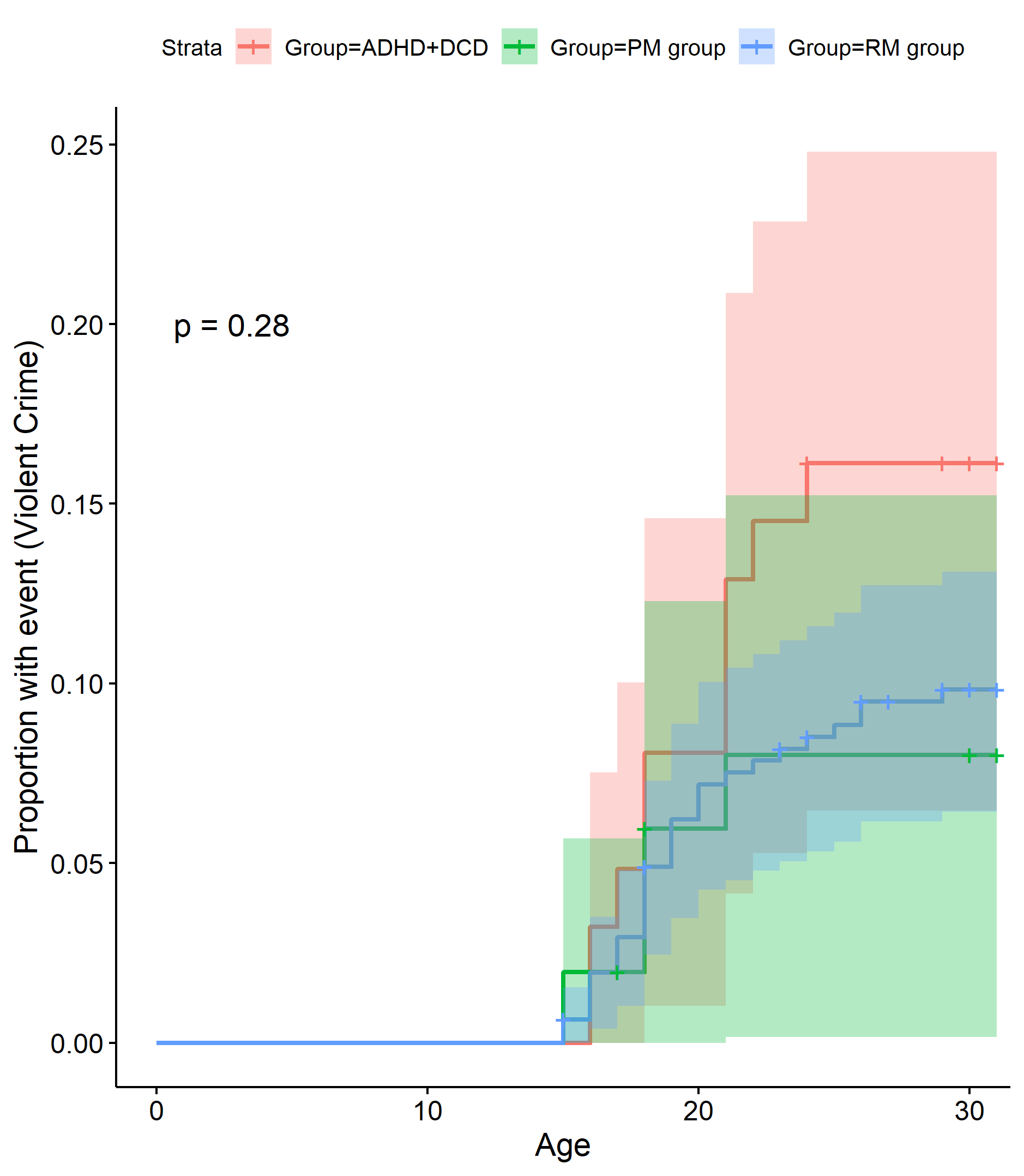
Depicted are Kaplan-Meier curves and overall significance of logrank test of difference across the three groups; ADHD+DCD (n=62), Population Matched group (PM group, n=51) and Registry Matched group (RM group, n=307). Censoring was due to emigration or death.

**Criminality Figure 2**
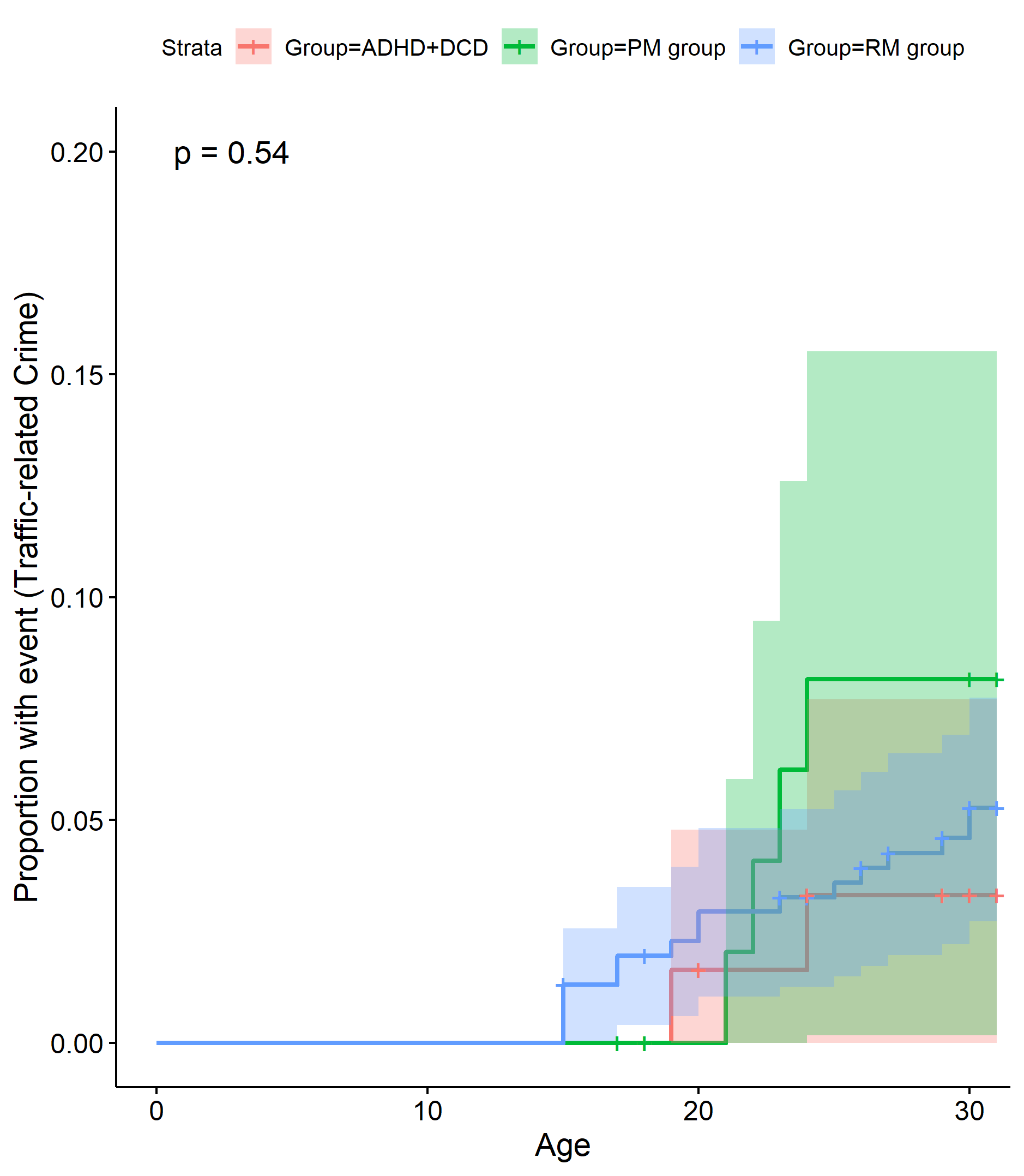


Depicted are Kaplan-Meier curves and overall significance of logrank test of difference across the three groups; ADHD+DCD (n=62), Population Matched group (PM group, n=51) and Registry Matched group (RM group, n=307). Censoring was due to emigration or death.

**Criminality Figure 3**
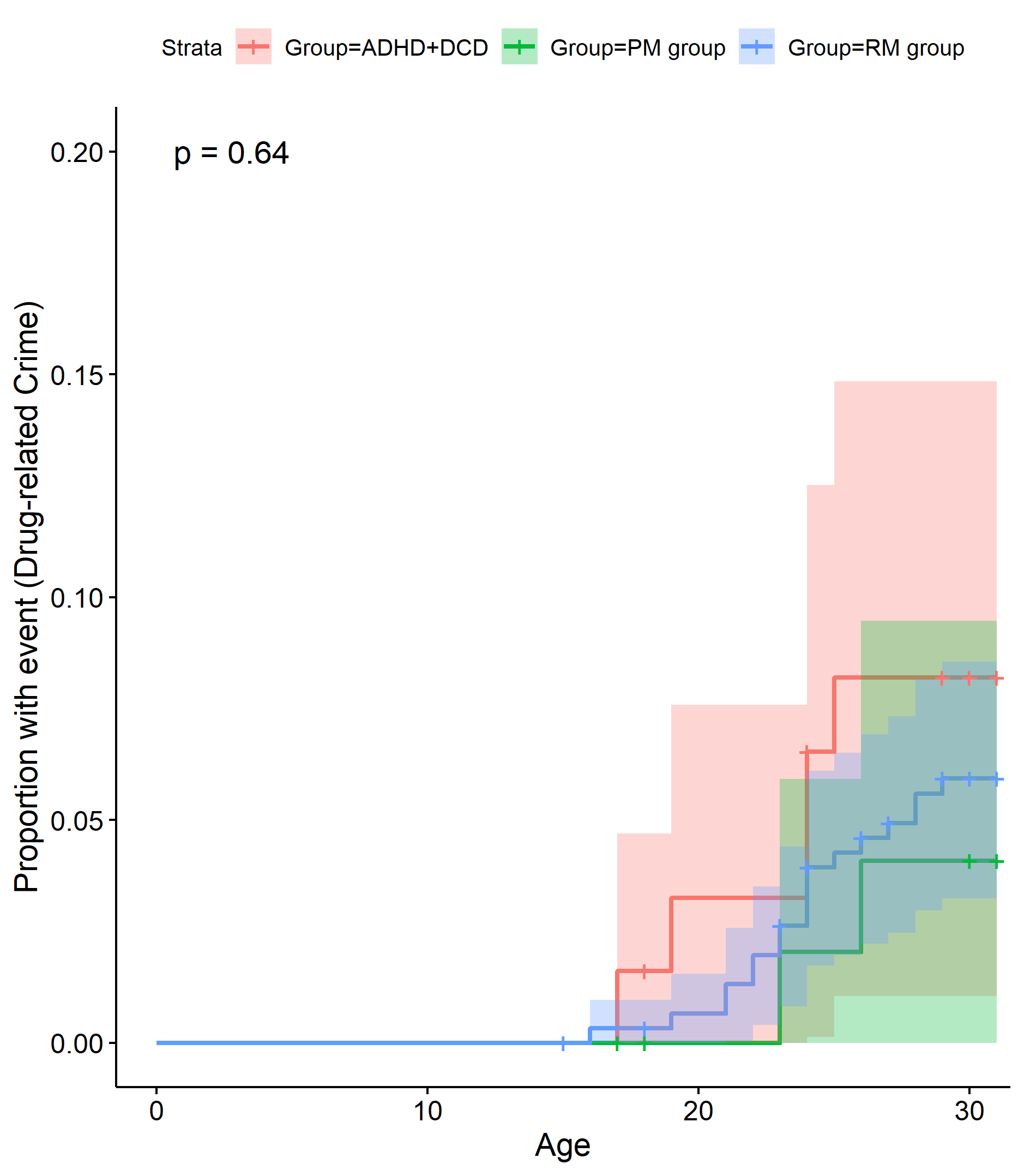


Depicted are Kaplan-Meier curves and overall significance of logrank test of difference across the three groups; ADHD+DCD (n=62), Population Matched group (PM group, n=51) and Registry Matched group (RM group, n=307). Censoring was due to emigration or death.

#### **Medical and Psychiatric Outcomes Figure 1**
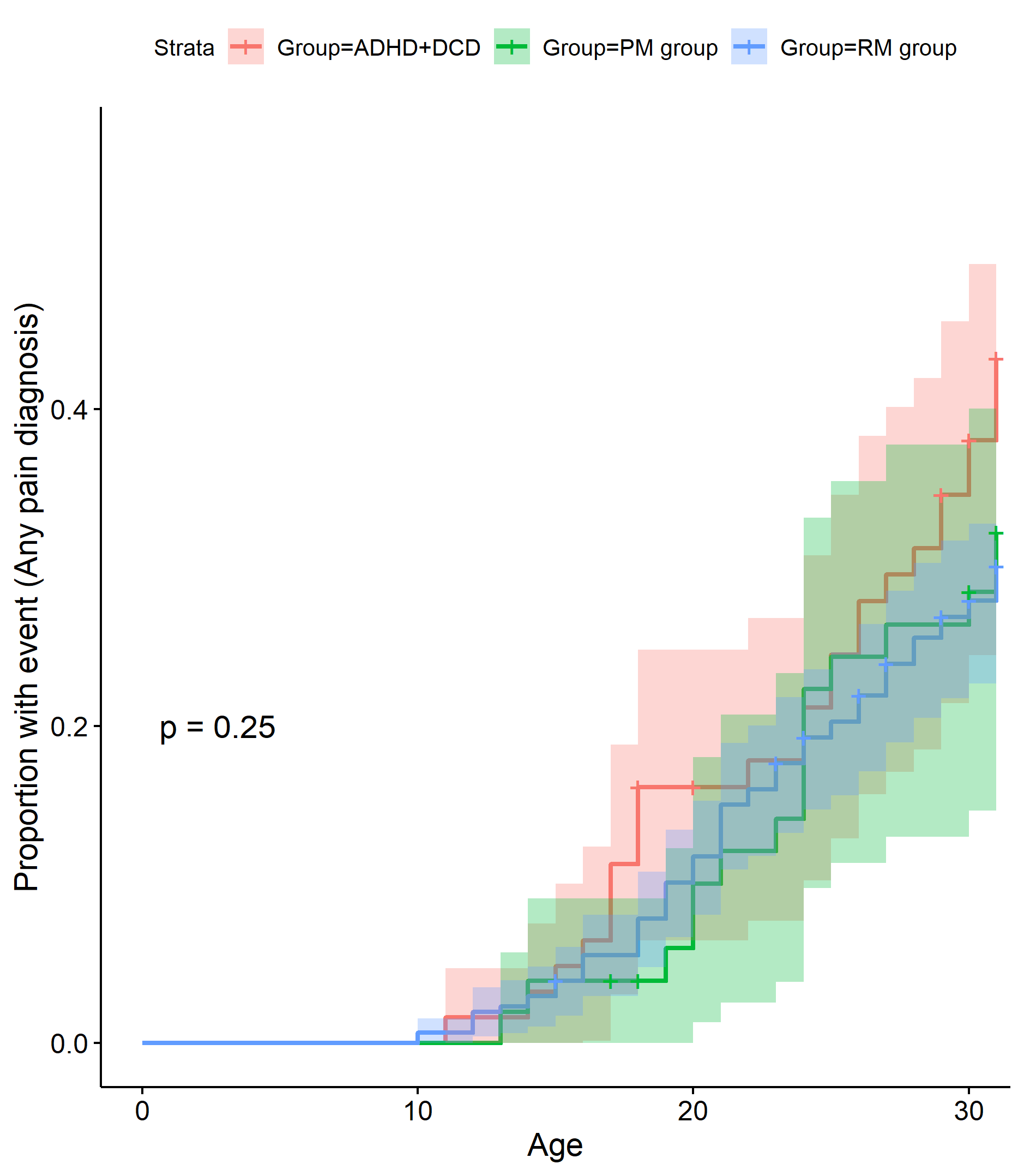


Depicted are Kaplan-Meier curves and overall significance of logrank test of difference across the three groups; ADHD+DCD (n=62), Population Matched group (PM group, n=51) and Registry Matched group (RM group, n=307). Censoring was due to emigration or death.

**Medical and Psychiatric Outcomes Figure 2**
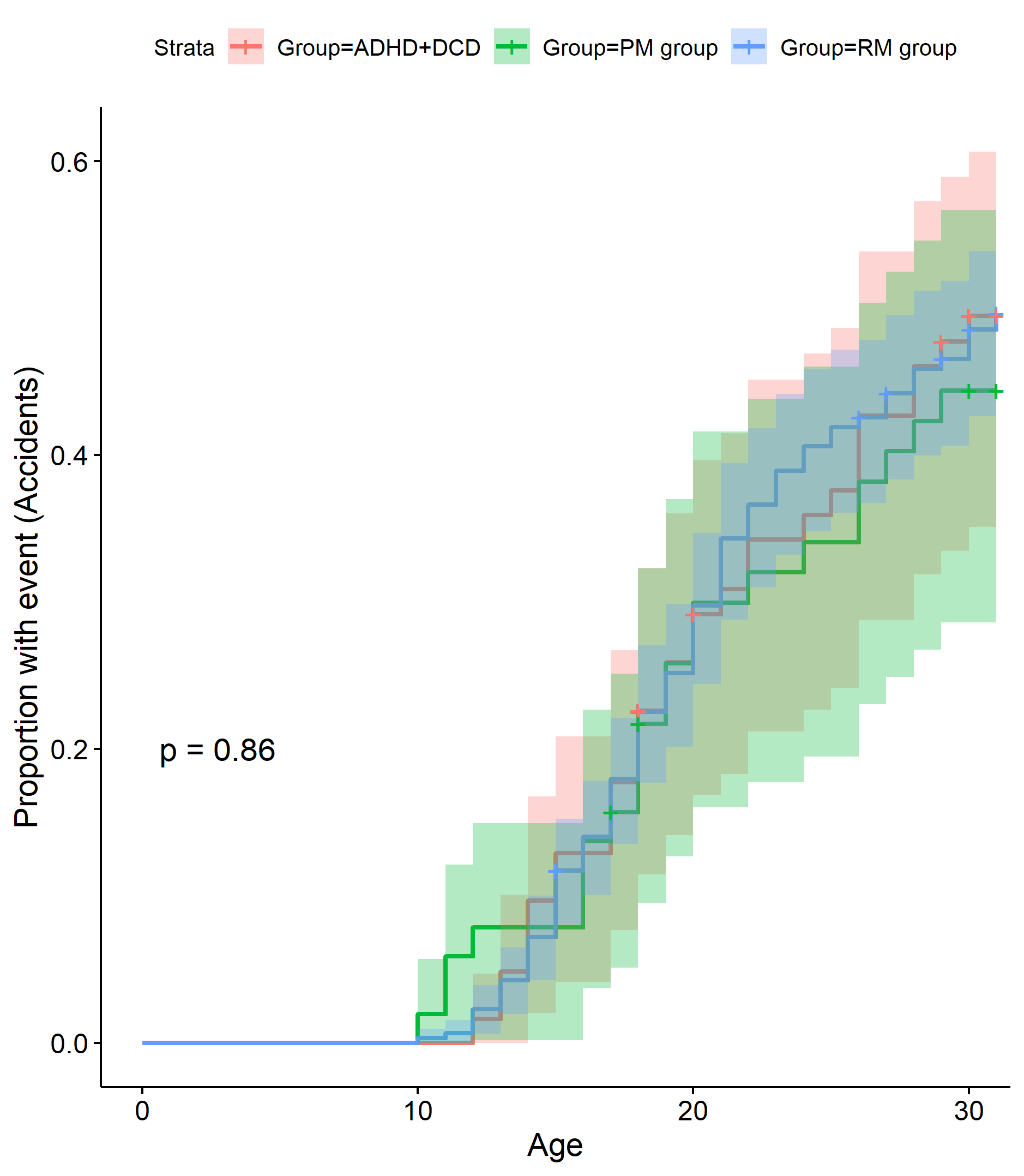


Depicted are Kaplan-Meier curves and overall significance of logrank test of difference across the three groups; ADHD+DCD (n=62), Population Matched group (PM group, n=51) and Registry Matched group (RM group, n=307). Censoring was due to emigration or death.

**Medical and Psychiatric Outcomes Figure 3**
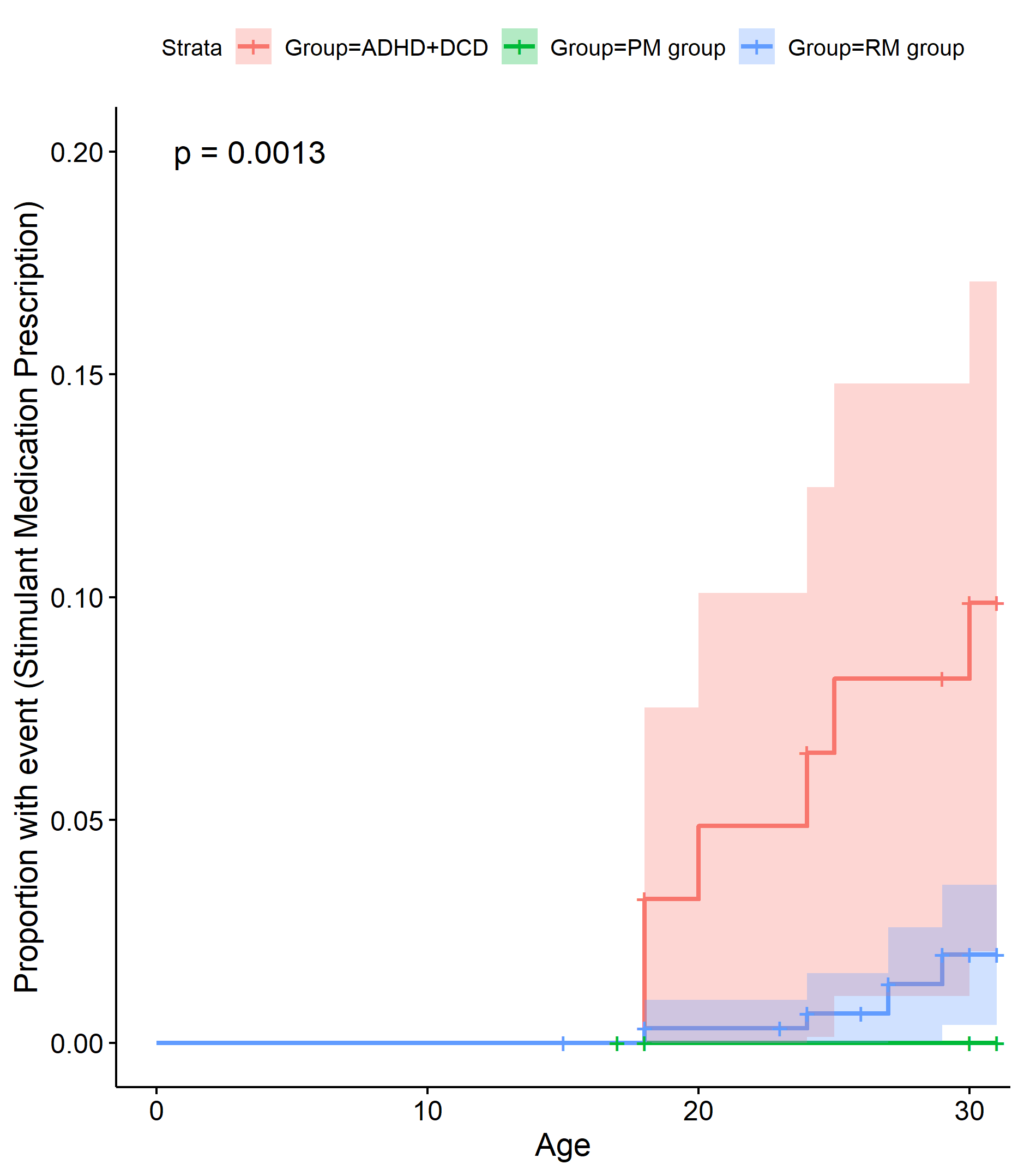
 Depicted are Kaplan-Meier curves and overall significance of logrank test of difference across the three groups; ADHD+DCD (n=62), Population Matched group (PM group, n=51) and Registry Matched group (RM group, n=307). Censoring was due to emigration or death.

**Medical and Psychiatric Outcomes Figure 4**
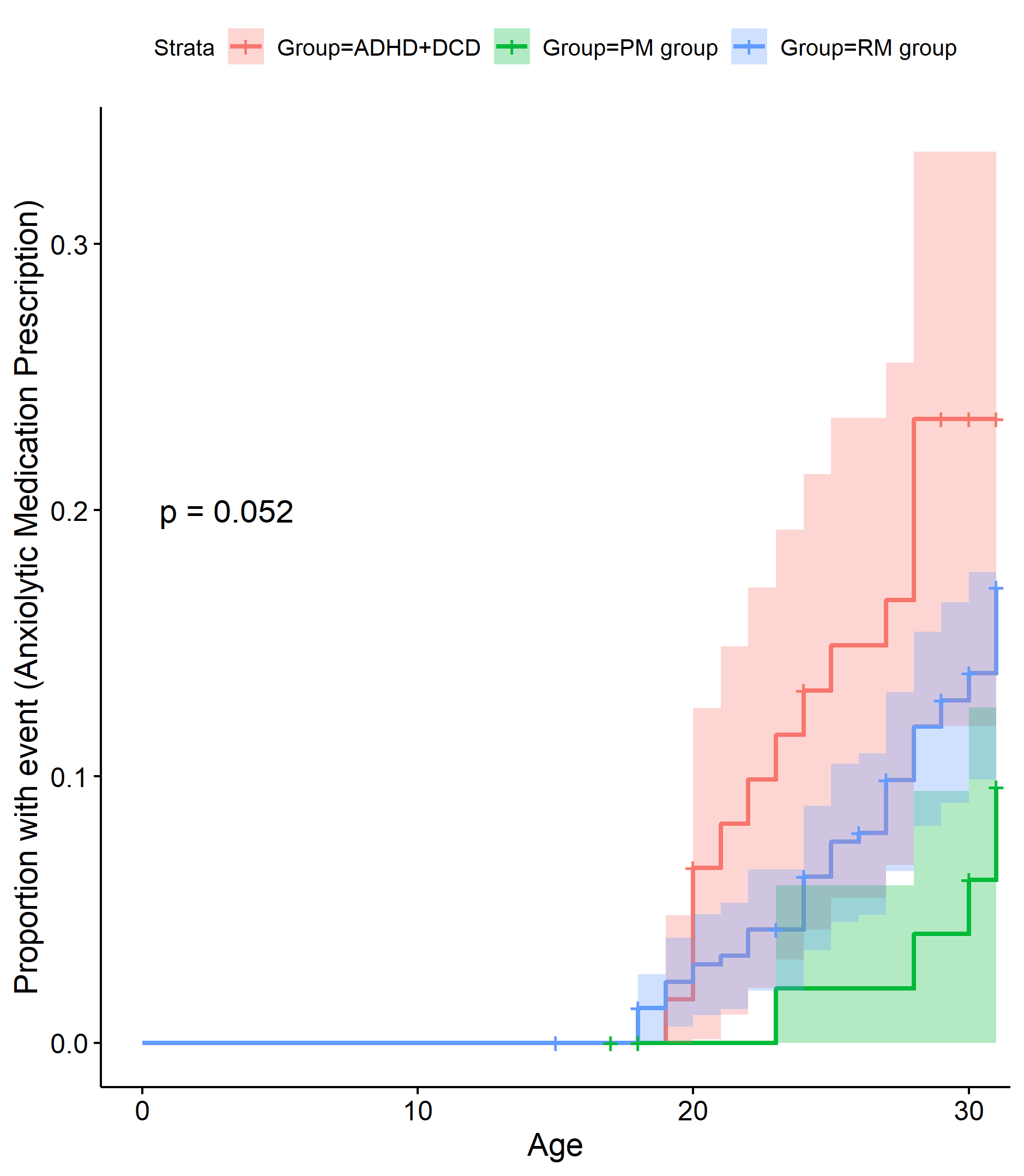


Depicted are Kaplan-Meier curves and overall significance of logrank test of difference across the three groups; ADHD+DCD (n=62), Population Matched group (PM group, n=51) and Registry Matched group (RM group, n=307). Censoring was due to emigration or death.

**Medical and Psychiatric Outcomes Figure 5**
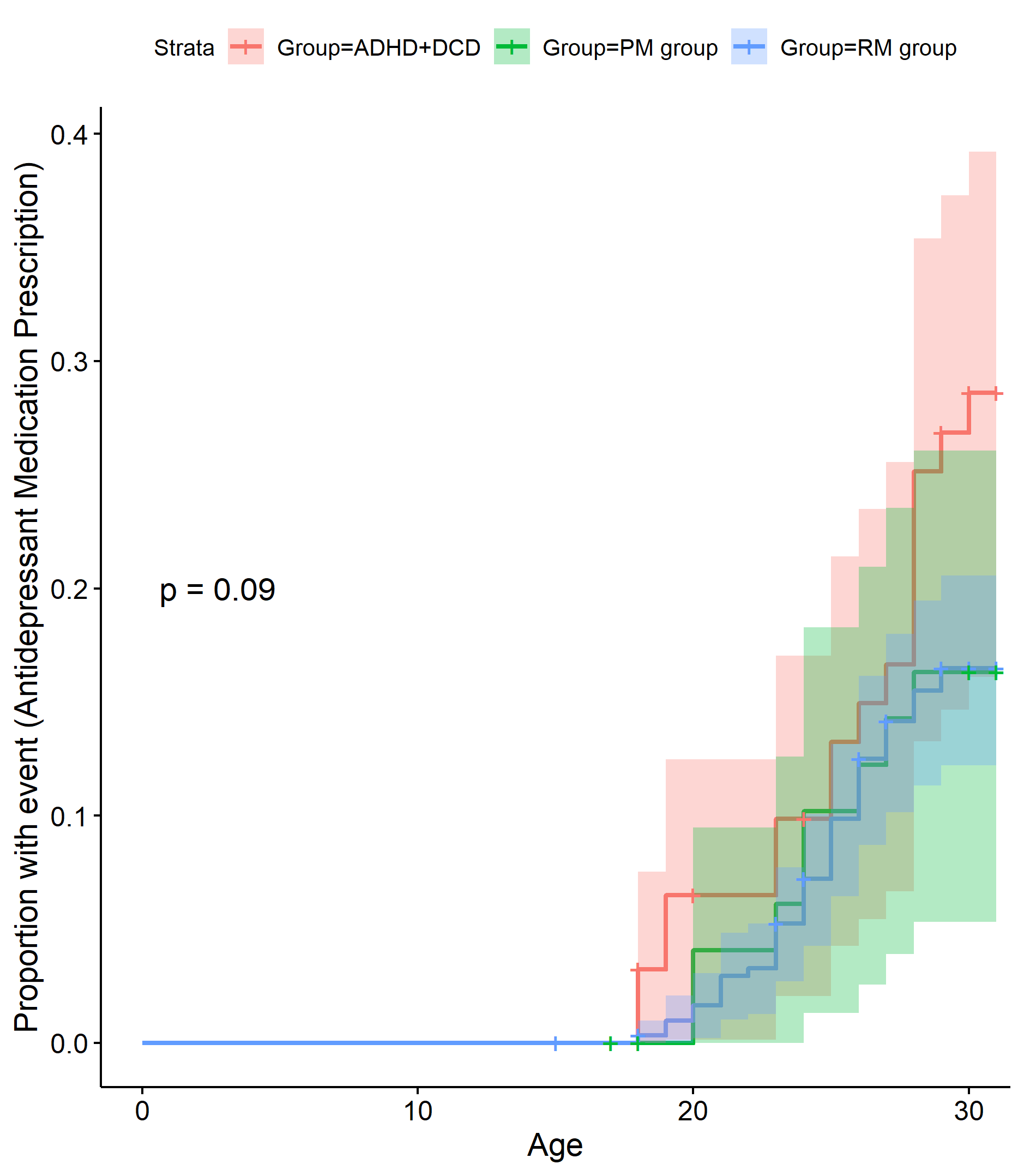
 Depicted are Kaplan-Meier curves and overall significance of logrank test of difference across the three groups; ADHD+DCD (n=62), Population Matched group (PM group, n=51) and Registry Matched group (RM group, n=307). Censoring was due to emigration or death.

**Medical and Psychiatric Outcomes Figure 6**
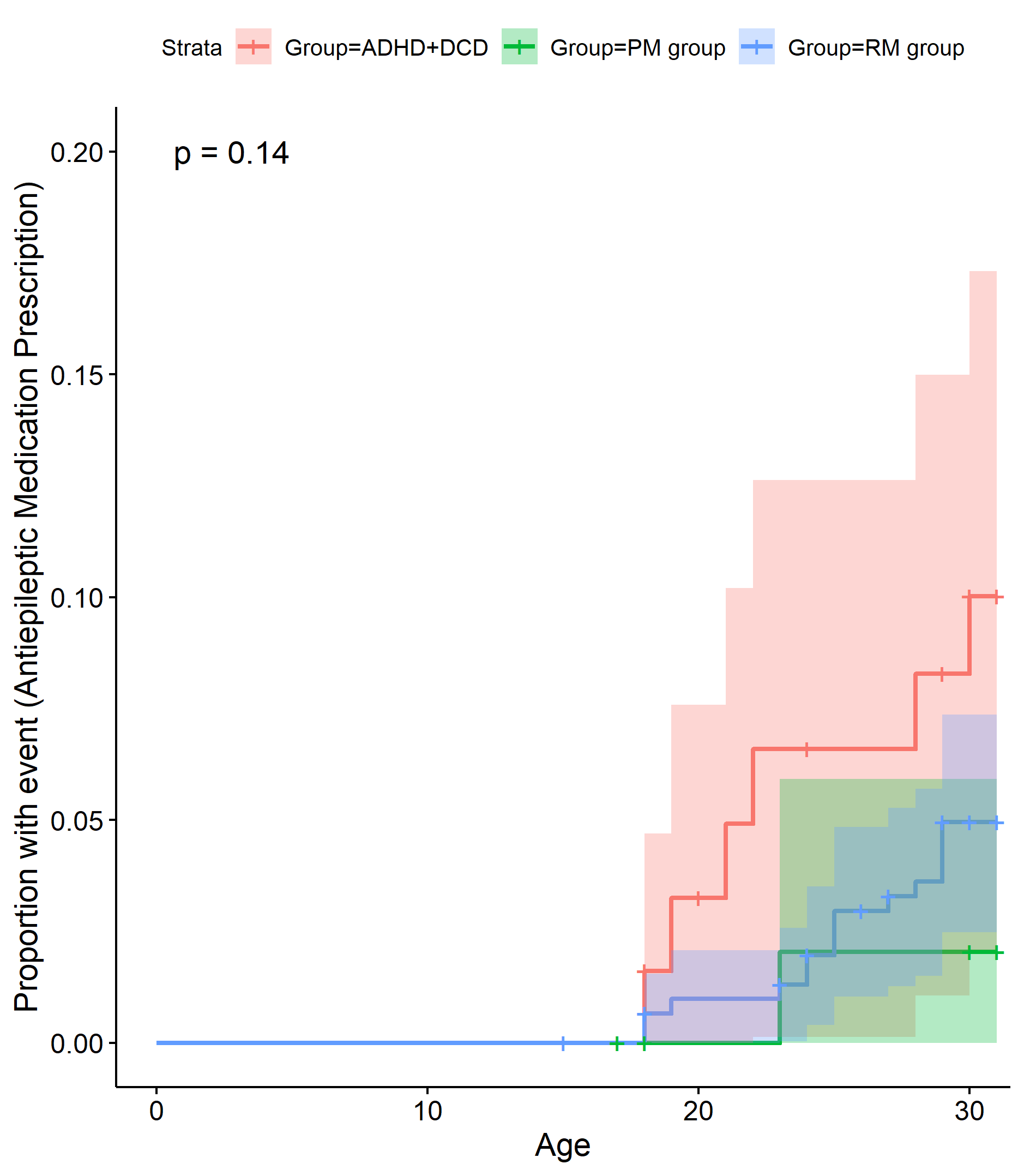


Depicted are Kaplan-Meier curves and overall significance of logrank test of difference across the three groups; ADHD+DCD (n=62), Population Matched group (PM group, n=51) and Registry Matched group (RM group, n=307). Censoring was due to emigration or death.

**Medical and Psychiatric Outcomes Figure 7**
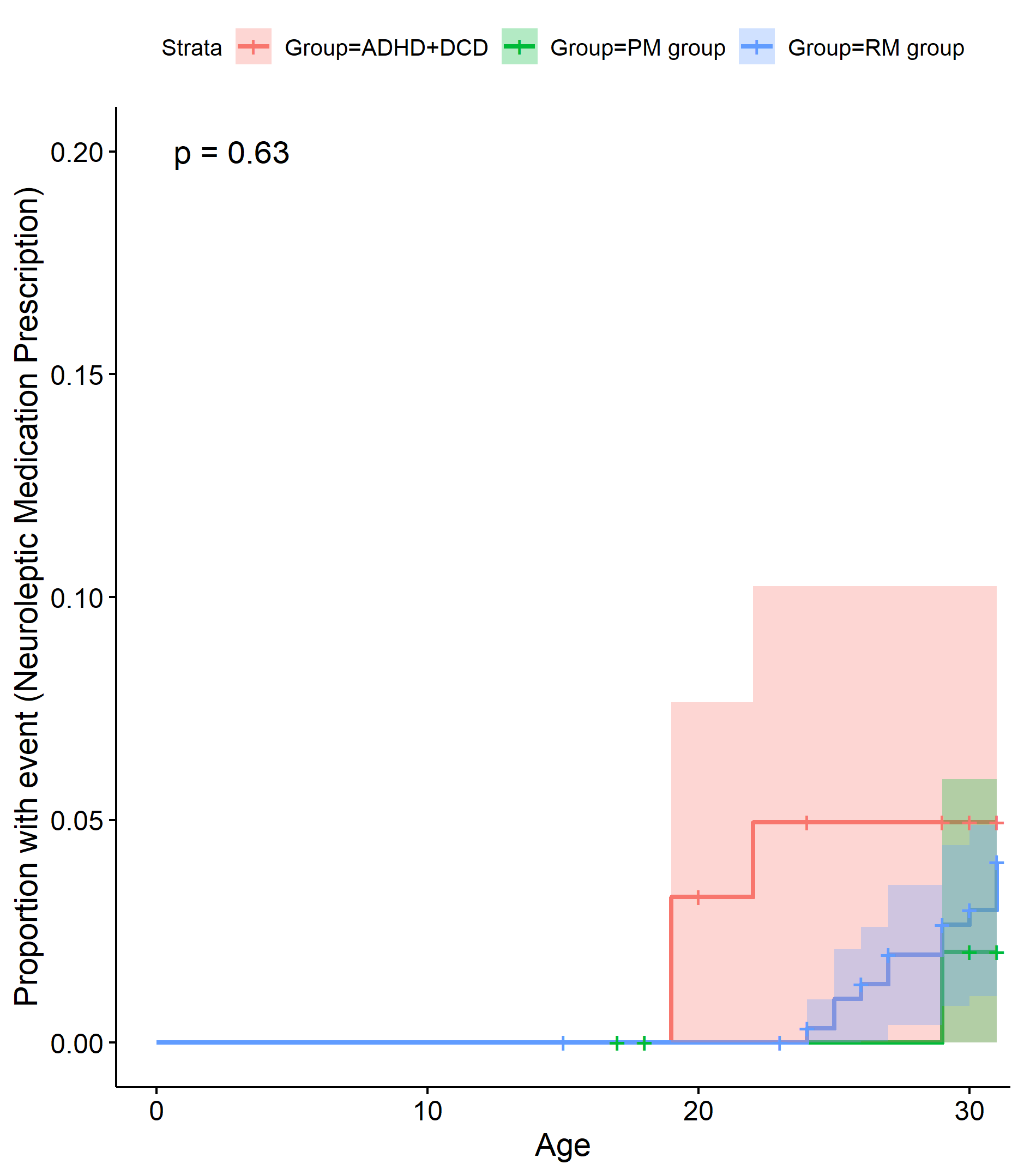


Depicted are Kaplan-Meier curves and overall significance of logrank test of difference across the three groups; ADHD+DCD (n=62), Population Matched group (PM group, n=51) and Registry Matched group (RM group, n=307). Censoring was due to emigration or death.
